# Association analysis of BSA-seq, BSR-seq, and RNA-seq reveals key genes involved in purple leaf formation in a tea population (*Camellia sinensis*)

**DOI:** 10.1093/hr/uhae191

**Published:** 2024-07-10

**Authors:** Yueqi Wang, Ji-Qiang Jin, Rui Zhang, Mengdi He, Liubin Wang, Zhuozhuo Mao, Min Gan, Liyun Wu, Liang Chen, Liyuan Wang, Kang Wei

## Abstract

Purple tea, rich in anthocyanins, has a variety of health benefits and is attracting global interest. However, the regulation mechanism of anthocyanin in purple tea populations has not been extensively studied. In this experiment, RNA-seq, BSA-seq, and BSR-seq were performed using 30 individuals with extreme colors (dark-purple and green) in an *F*_1_ population of ‘Zijuan’ and ‘Jinxuan’. The results show that 459 genes were differentially expressed in purple and green leaves, among which genes involved in the anthocyanin synthesis and transport pathway, such as *CHS*, *F3H*, *ANS*, *MYB75*, *GST*, *MATE*, and *ABCC,* were highly expressed in purple leaves. Moreover, there were multiple SNP/InDel variation sites on chromosomes 2 and 14 of the tea plant, as identified by BSA-seq. The integrated analysis identified two highly expressed genes (*CsANS* and *CsMYB75*) with SNP/InDel site variations in the purple tea plants. By silencing leaves, we proved that *CsMYB75* could positively regulate anthocyanin accumulation and expression of related structural genes in tea plants. A 181-bp InDel in the *CsMYB75* promoter was also found to be co-segregating with leaf color. The results of this study provide a theoretical reference for the molecular mechanism of anthocyanin accumulation in purple tea plants and contribute to the creation of new tea cultivars with high anthocyanin content.

## Introduction

The tea plant originated in China and is extensively cultivated worldwide as a valuable cash crop. The leaves of regular tea plants are green, while purple tea emerged through long-term natural evolution. Purple tea, known for its high level of anthocyanins, is made from the buds of purple tea plants and is a natural health beverage rich in secondary metabolites of flavonoids [1]. Studies have shown that purple tea extract can regulate the intestinal microbiota and improve obesity and metabolic disorders caused by high fat intake [2]. Its anti-inflammatory and anti-proliferative properties may also be used for cancer prevention [3]. In addition, purple tea has a strong antioxidant capacity, which shows benefits in anti-aging [4], neuroprotection [5], and other aspects. Taking these properties together, the purple tea plant has high research value as a potential resource of natural raw materials for functional tea beverages.

Anthocyanins belong to the flavonoids, a class of natural pigments widely distributed in nature, and their accumulation is the primary cause of purple coloration in plants [6]. Studies have shown that the significant upregulation of structural genes, such as anthocyanidin synthase (*ANS*), dihydroflavonol reductase (*DFR*), and flavonoid-3-*O*-glycosyltransferase (*UFGT*), and key regulatory genes like *MYB* and *bHLH* can promote the transfer of metabolites towards anthocyanin biosynthesis [7–9]. Furthermore, He *et al.* isolated the transcription factor gene *CsMYB6A* in purple leaves, and its overexpression could activate the expressions of flavonoid-related structural genes [10]. Sun *et al.* activated the transcription factor gene *CsAN1*, which specifically upregulated the late anthocyanin biosynthesis genes, leading to the ectopic accumulation of purple pigment [11]. Wei *et al.* found that the transcription factor gene *CsMYB75* and the glutathione transferase gene *CsGSTF1* are related to the purple leaf color phenotype [12, 13]. These studies provide valuable resources for the evolution and breeding of purple tea plants. However, the complex genetic background of various tea cultivars increases the complexity of pigment biosynthesis, and the results obtained for a single-purple cultivar as a research object cannot be generally applied to tea plant populations. The precise exploration of the molecular mechanism of purple formation therefore remains an ongoing objective.

**Figure 1:**
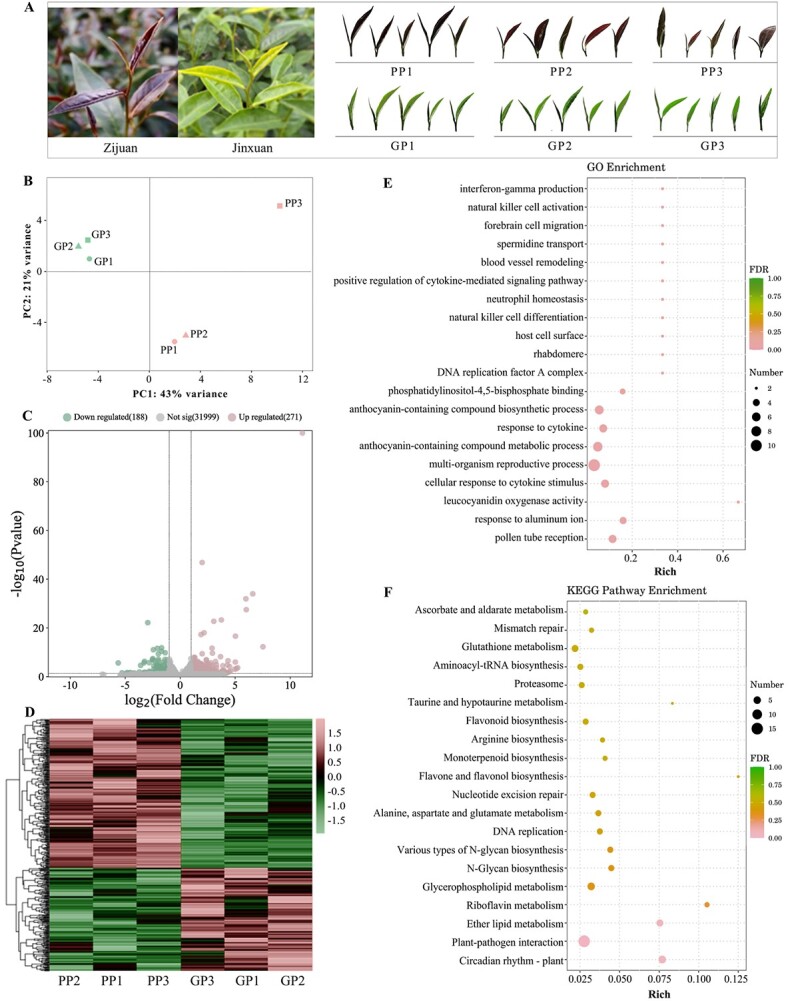
RNA-seq analysis of green and purple tea samples. **A** Phenotypes of parents ZJ and JX and their offspring used in the experiment. **B** PCA analysis of RNA-seq results of green and purple tea samples. **C** Clustering heat map of DEGs, normalization. **D** Volcano plot of DEGs. **E** Bubble chart of GO enrichment of DEGs. **F** Bubble chart of KEGG pathway enrichment of DEGs.

In recent years, various combinations of different high-throughput technologies, such as quantitative trait locus (QTL) analysis, bulked segregant RNA (BSR) analysis, bulked segregant analysis (BSA), and RNA-seq, have been used to explore the potential mechanism of plant color and more accurately locate related genes. Relative studies on oilseed rape red flower [14] and barley black skin [15] show that the combinations of these methods are highly efficient and powerful in the identification of key genes. Therefore, the above multi-technique combined analysis also presents a new approach for identifying potential genes associated with anthocyanin accumulation in tea plants.

In this study, we obtained a hybrid population of purple tea cultivar ‘Zijuan’ (ZJ) and green leaf tea cultivar ‘Jinxuan’ (JX), from which a total of 30 tea individuals with extreme leaf colors (15 dark-purple leaf individuals and 15 green leaf individuals) were selected (Fig. 1A). BSA-seq and BSR-seq were used to reveal the candidate regions and gene mutation sites associated with purple leaf formation in tea plants, and RNA-seq was used to identify the differentially expressed genes (DEGs) among different leaf colors. The integrated analysis aimed to find the key genes and variation patterns for purple leaf formation in the tea population.

## Results

### Differentially expressed genes between purple and green individuals in the tea population

A hybrid population of purple tea cultivar ZJ and green leaf cultivar JX, which includes 127 individuals, was used in this study. This population varied in their leaf coloration from being totally green (including 18 individuals) to dark purple (32 individuals) (Supplementary Data Fig. S1). A total of 30 tea individuals with extreme leaf colors (15 dark purple and 15 green leaf individuals) were used for subsequent analysis. To identify genes potentially associated with the leaf color phenotype, autumn samples were subjected to RNA-seq analysis. More than 42 011 640 raw reads were obtained and 39 532 308 clean reads were obtained after removing the adapter and low-quality reads. The sequencing shows that the RNA-seq result was of high quality (Q30$\geqslant$ 93.57%, Q20 $\geqslant$ 97.82%) (Supplementary Data Table S1), and >90.50% of clean read segments were successfully mapped to the ‘Tieguanyin’ reference genome (Supplementary Data Table S2). Principal component analysis (PCA) revealed that all samples could be grouped into two distinct clusters, with a significant difference between the groups (Fig. 1B). Therefore, it is believed that the quality of the samples is reliable and can be analyzed for subsequent study.

To further explore the genes influencing the purple leaf phenotypes, we used |log_2_ (fold change)| > 1 and *P* < 0.05 as the criteria for identifying DEGs with the green samples as the control. A total of 459 DEGs were obtained, of which 188 genes were downregulated and 271 genes were upregulated in the purple tea samples (Fig. 1C and D). Based on the sequence comparison and annotation results, these DEGs were restored to the pathway analysis using Gene Ontology (GO) and Kyoto Encyclopedia of Genes and Genomes (KEGG), and the top 20 term entries with the most significant overall enrichment were selected for display (Fig. 1E and F). Among them, the biosynthetic and metabolic processes of anthocyanin-containing compounds, as well as leucocyanidin oxygenase activity, are significantly enriched according to GO analysis. Flavonoid biosynthesis, flavone and flavonol biosynthesis, and glutathione metabolism are significantly enriched according to KEGG analysis. These pathways related to anthocyanin accumulation may contribute to the differences in leaf color.

**Figure 2:**
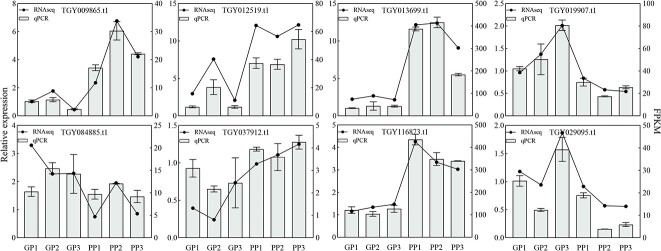
qRT–PCR analysis of genes in all samples. GP and PP represent green and purple tea plants.

To validate the RNA-seq results, eight genes potentially associated with anthocyanin biosynthesis or other important biosyntheses were selected for qRT–PCR analysis (Fig. 2). These included ANS (*TGY116873.t1*), MYB transporter (*TGY012519.t1*, *TGY084885.t1*), GST transferase (*TGY013699.t1*), UDP-glycosyltransferase (*TGY019907.t1*), ABC transporter (*TGY037912.t1*), phenolic glucoside malonyltransferase (*TGY009865.t1*), and hydroquinone glucosyltransferase-like (*TGY029095.t1*). The results show that the gene expression trend of the qRT–PCR was highly consistent with that of RNA-seq, suggesting that the RNA-seq result was reliable in identifying DEGs.

### BSA-seq analysis of purple and green tea samples

Based on the Illumina NovaSeq sequencing platform, paired-end sequencing was performed on two extreme mixed pools, yielding a total of 56 109 638 812 and 47 710 662 150 bp of raw data (Supplementary Data Table S3). The quality evaluation shows that the GC contents of these reads were 40.45–40.46%, with Q20 ≥ 97.27% and Q30 ≥ 92.98%, indicating high sequencing data quality. After removing the splices and low-quality reads, we obtained 358 640 732 and 304 889 406 reads from green tea and purple tea, respectively (Supplementary Data Table S4). These data were subjected to further analysis.

**Figure 3:**
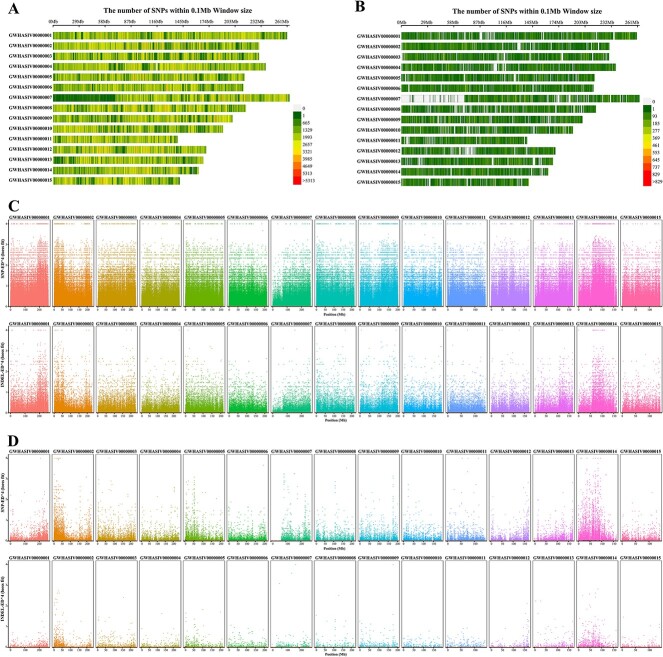
BSA-seq and BSR-seq analysis. **A** Distribution of SNPs on chromosomes by BSA-seq analysis. **A** Distribution of SNPs on chromosomes by BSR-seq analysis. **C** Map of ED values on chromosomes based on SNPs and InDels by BSA-seq analysis. **D** Map of ED values on chromosomes based on SNPs and InDels by BSR-seq analysis.

The obtained reads were mapped to the ‘Tieguanyin’ reference genome, and the results show that the sample had a high localization rate (99.53–99.67%) and uniform random coverage of the reference genome (Fig. 3A). Furthermore, a total of 51 071 018 single nucleotide polymorphisms (SNPs) and 2 293 034 insertion-deletions (InDels) were obtained after screening and annotation (Supplementary Data Table S5). To identify the linked BSA regions closely related to leaf color, the euclidean distance (ED) value was used to calculate the frequency distance of each mutant between the two mixed pools, reflecting the linkage strength between the marker and the target region for localization. A total of 1554 genes were found, among which the SNPs were mainly distributed in chromosome 14 (57 542 636–13 088 670 645 bp), while the InDels were mainly distributed in chromosomes 1 (201 720 575–213 703 593 bp), 2 (17 325 640–24 134 324 bp), and 14 (58 360 707–109 194 469 bp) (Fig. 3C).

### BSR-seq analysis of purple and green tea samples

BSR-seq generated 19 471 240 200 and 20 702 142 600 bp of raw data in the two extreme mixing pools, respectively (Supplementary Data Table S6), and these data were of high quality (GC 45.49–46.39%, Q20 ≥ 97.91%, and Q30 ≥ 93.81%). After the removal of the splicing and low-quality reads, 126 664 798 and 134 283 326 reads were obtained from green tea and purple tea, respectively (Supplementary Data Table S7), and further analyzed.

The obtained reads were also mapped to the ‘Tieguanyin’ reference genome, which showed that the sample had a high localization rate (99.51–99.52%) and uniform random coverage of the reference genome (Fig. 3B). After screening and annotation, a total of 1 052 862 SNPs and 97 338 InDels were obtained (Supplementary Data Table S8). The ED values were calculated to reflect the linkage strength between the marker and the target region for localization, and 1387 genes were found. Among them, the SNPs were mainly distributed in chromosomes 5 (0–10 538 546 bp, 46 191 998–60 513 830 bp) and 14 (0–171 041 bp, and 12 671 613–101 253 520 bp), and the InDels were mainly distributed in chromosomes 2 (9 082 319–11 033 742 bp, 11 061 869–14 938 348 bp), 5 (0–9 456 268 bp), 12 (162 047 230–170 056 323 bp), 13 (167 277 146–167 397 723 bp), and 14 (20 191 554–30 474 215 bp, 36 030 033–67 181 622 bp, and 82 937 505–110 378 151 bp) (Fig. 3D).

### Association analysis of BSA-seq, BSR-seq and RNA-seq data to identify the important genes involved in purple tea leaf formation

**Figure 4:**
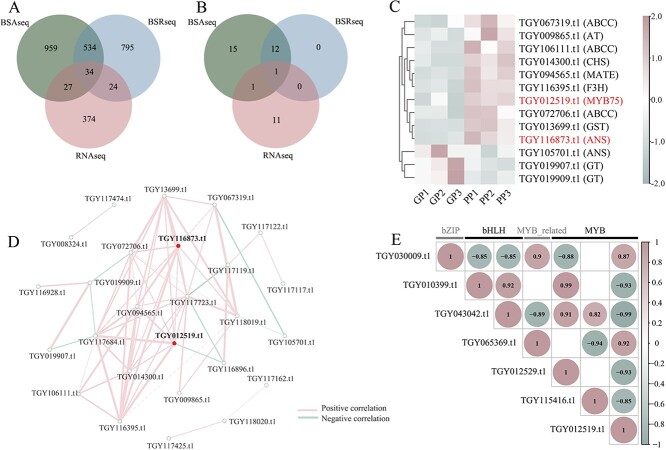
Analysis of candidate genes for BSA-seq, BSR-seq, and RNA-seq combined. **A** Venn diagram of genes. **B** Venn diagram of 40 genes in flavonoid biosynthesis. **C** Clustering heat map of 13 DEGs in flavonoid biosynthesis, normalization. **D** Correlation network of 40 genes in flavonoid biosynthesis. Line thickness indicates correlation size, which ranges from 0.8 to 1.0; the thicker the line，the larger the correlation. **E** Correlation of other transcription factors with *CsMYB75* (*TGY012519.t1*).

Previously, separate analysis of BSR-seq, BSA-seq, and RNA-seq was performed and the candidate genes involved in purple tea leaf formation were preliminarily identified (Figs 1 and 2). To further identify genes associated with leaf color, we integrated the analysis results of BSR-seq, BSA-seq, and RNA-seq, and identified a total of 2747 genes in the three groups (Fig. 4A). After functional annotation, it was determined that 40 genes associated with the biosynthesis pathway of anthocyanins/flavonoids were either differentially expressed or underwent site mutations in this population (Fig. 4B and D). Among them, Venn diagram analysis revealed a candidate key gene, *TGY116873.t1* (*CsANS*), shared in all three groups, which is highly expressed in purple leaves and exhibits multiple site variations at the RNA and DNA levels on chromosome 14. In addition, given the limitation of BSR-seq in detecting mutations occurring in the gene regulatory regions, a key gene, *TGY012519.t1* (*CsMYB75*), was found to be shared by both BSA-seq and RNA-seq results, which is highly expressed in purple leaves and has locus variation on chromosome 2. Besides, based on transcriptome data, there were 11 genes, including *CHS*, *F3H*, *GT*, *AT*, *GST*, *MATE*, and *ABC*, differentially expressed in the purple and green tea samples and potentially involved in the synthesis and transport pathway of anthocyanins in tea plants (Fig. 4C).

As *CsMYB75* is an important transcription factor identified in our previous studies, how it operates in a complex with other partners is an essential question to answer. Therefore, we expanded our search to identify any additional candidate genes from the transcription dataset. Correlation analysis shows that six transcription factor genes were significantly correlated with *CsMYB75*, belonging to the bHLH, MYB, and IZIP families (Fig. 4E). Of these, two genes (*TGY065369.t1* and *TGY030009.t1*) were significantly and positively correlated with *CsMYB75*, while four genes (*TGY010399.t1*, *TGY043042.t1*, *TGY115416.t1*, and *TGY012529.t1*) were negatively correlated, suggesting the regulation system is complex.

### Validation of the function of *CsMYB75* in tea plants

**Figure 5:**
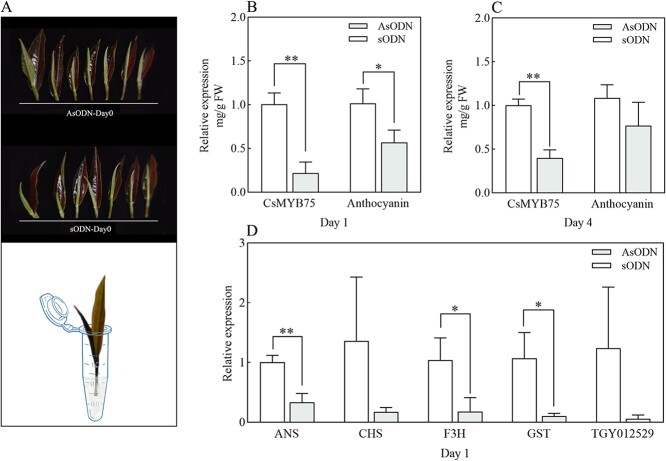
Analysis of AsODN-*CsMYB75* experimental results in tea leaves. **A** Tea shoots of ZJ used in the *CsMYB75* expression experiment, were recorded on day 0. **B**–**D** Anthocyanin content, expression levels of *CsMYB75*, and other related genes on different feeding days with AsODN–sODN solution. Differences were analyzed by the *t*-test (^*^P < 0.05, ^**^P < 0.01).

To delve deeper into the importance of *CsMYB75* in anthocyanin accumulation, antisense oligonucleotides (AsODNs) were used to silence *CsMYB75* in the purple tea cultivar ZJ (Fig. 5A). After feeding AsODN–sODN solution for 1 and 4 days, the expression of *CsMYB75* was significantly decreased compared with that of the control. The anthocyanin content also showed a decreasing trend, especially on the first day (Fig. 5B and C). We further selected several structural genes and a transcription factor potentially involved in anthocyanin accumulation for quantitative analysis on the first day (Fig. 5D). The results show that the expressions of *ANS*, *F3H*, and *GST* were significantly decreased after *CsMYB75* silencing, indicating the important role of *CsMYB75* in controlling the downstream genes.

### Analysis of the promoters of *CsMYB75* and development of a functional marker

**Figure 6:**
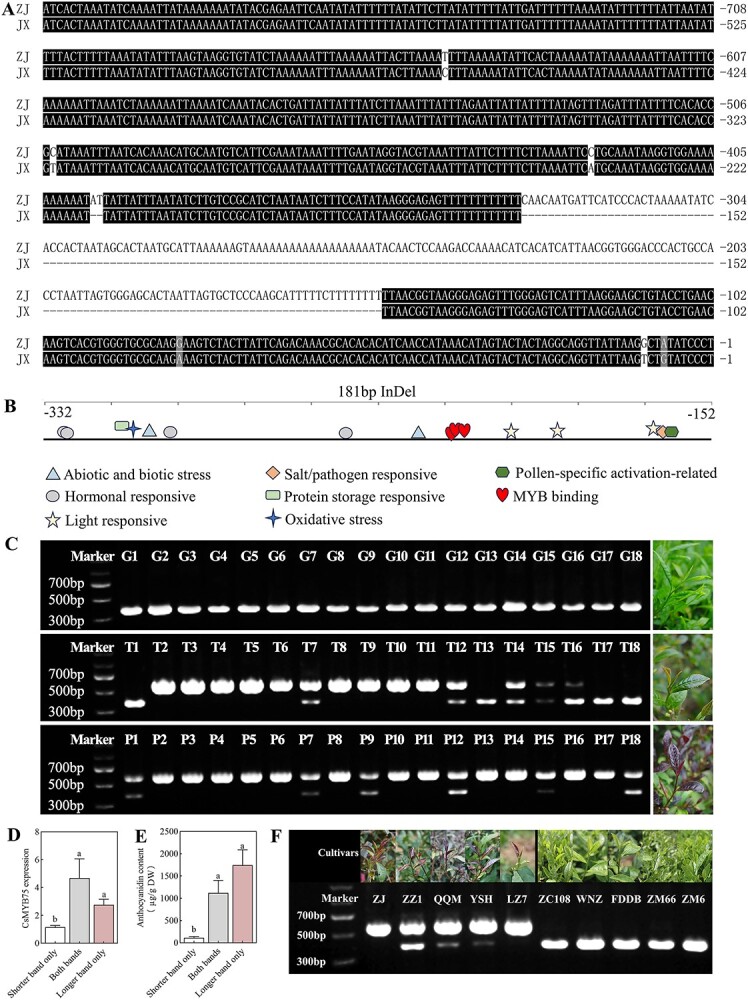
Analysis of promoters of *CsMYB75*. **A** Comparison of the promoter sequences of *CsMYB75* in ZJ and JX. **B** Distribution of *cis*-acting elements on the *CsMYB75* promoter sequences. **C** Functional marker development validation in the *F*_1_ population. **D**, **E** Expression of *CsMYB75* and anthocyanin contents in the *F*_1_ population. Different letters mean significant differences. **F** Functional marker development validation in five purple leaf and five green leaf tea cultivars.

Since *CsMYB75* plays an important role in anthocyanin biosynthesis, a key question to be answered is why it can be highly expressed in purple tea individuals. Therefore, the *CsMYB75* promoters of the purple tea cultivar ZJ and green leaf cultivar JX were cloned and compared. Sequence alignment shows that their promoters are highly similar except for a 181-bp InDel between −152 and −332 (with the start codon ATG as the starting site) (Fig. 6A). Further promoter prediction of this InDel shows that it contains various *cis*-acting elements, such as light, stress, and hormone-responsive elements. In addition, it also contains three MYB transcription factor recognition and binding sequence sites (MYBCORE, MYBCOREATCYCB1, MYB2CONSENSUSAT) (Fig. 6B, Supplementary Data Table S9).

To verify its correlation with the purple leaf phenotype, an InDel marker based on this 181-bp insertion was developed and tested in the *F*_1_ population. Each green leaf individual contains only one shorter band (~400 bp, without the 181-bp insertion) (Fig. 6C), while most slight purple individuals (transition color) have a longer band (~600 bp). Interestingly, all dark purple individuals contain the longer band. We further analyzed the expression of *CsMYB75* and anthocyanin content in the *F*_1_ population. Clearly, individuals with a shorter band only had the lowest expression of *CsMYB75* and lowest content of anthocyanin, while those in the individuals with a longer band were significantly increased (Fig. 6D and E). To further investigate whether this InDel is related to the purple bud trait in other tea germplasms, we analyzed the performance of this marker in different tea cultivars (including ZJ, four other purple leaf tea cultivars and five green leaf cultivars). The results were consistent with that in the *F*_1_ population (Fig. 6F). These results indicate that the 181-bp insertion could significantly enhance the expression of *CsMYB75*, thereby affecting the formation of purple tea.

## Discussion

As tea is a cash crop for leaves, the color of tea plays an important role in the phenotype and quality. With the deepening of research, the combination of various high-throughput techniques to accurately screen and identify key genes affecting leaf color has become a hot topic, among which metabolomics and transcriptome analysis are the most widely used methods [16, 17]. On this basis, in the field of plant leaf color, DNA methylation, QTL, BSA, and other technologies continue to emerge [14, 15, 18]. However, only QTL can be seen in limited reports on the purple phenotype of tea, which was used to locate gene regions and then to further targeted genes through transcriptome data analysis [12, 19]. Therefore, in this experiment, BSA and BSR technologies combined with transcriptome technology were innovatively used to study the purple leaf trait of tea.

### Structural genes of anthocyanin synthesis were highly expressed in purple tea samples

Anthocyanins are natural pigments found in plants that play a crucial role in determining the color phenotype. The expressions of key genes involved in regulating anthocyanin synthesis, such as *C4H*, *CHS*, *F3H*, and *ANS*, were shown to be different in various tea cultivars [6, 8–11]. However, most previous studies have been conducted on a single purple cultivar, and there are few reports on experiments using diverse tea populations as materials. To investigate a more accurate mechanism for regulating anthocyanin in tea plants, experiments are carried out using a progeny population of ZJ and JX established by our research group. According to the RNA-seq results, there were 459 DEGs between the purple and green tea samples (Fig. 1). Among them, *CHS*, *F3H*, and *ANS*, involved in anthocyanin synthesis, were highly expressed in purple leaves. These genes not only provide intermediates for anthocyanin synthesis but also contribute to the synthesis of other flavonoids.

ANS is a key enzyme at the end of the anthocyanin biosynthetic pathway, and its high expression level in purple tea leaves enhances the accumulation of anthocyanins, as widely reported. In this study, the highly expressed *ANS* in purple leaves was the unique gene found to have site mutations at both DNA and RNA levels, which further demonstrates its importance in anthocyanin synthesis in the purple tea samples. However, the variation of *ANS* has not been studied in tea plants, while its homologs were widely reported in other plants. In *Punica granatum* without anthocyanins, an InDel site was found in the coding region that was completely linked to the white phenotype, leading to the non-transcription of *ANS* [20]. In *Gentiana triflora*, there was a 4-bp nucleotide deletion in the exon of *ANS1*, resulting in a premature termination codon and gene inactivation [21], and so on. Taken together, these data point to *ANS* as a promising gene for anthocyanin modification, warranting further investigation into the variation.

UFGT is the last enzyme in the anthocyanin biosynthesis pathway and plays a crucial role in maintaining the stability and water solubility of anthocyanins. In this study, two differentially expressed UFGT genes were identified as *CsUTG78A14* (*TGY19907.t1*) and *CsUTG78A15* (*TGY19909.t1*), which have been reported to participate in flavonol glycosylation [22, 23]. *CsUTG78A15* was reported to be involved in eugenol glucosylation [24]. A recent analysis confirmed that *CsUTG78A15* is also responsible for anthocyanin galactosylation [25], while the expression of this gene was low in the purple leaves in our experiment. Given that *CsUTG78A15* is involved in multiple metabolic regulations, we believe that its overall contribution to the purple phenotype is small, and it may be more involved in flavonol glycosylation than in anthocyanin production. In addition, acylation is an important modification of anthocyanins that contributes to stable coloration. Acyl residues are an important factor in anthocyanin transport by MATE proteins [26]. We recently discovered that a gene encoding phenolic glucoside malonyltransferase (*TGY009865.t1*) and a MATE gene (*TGY094565.t1*) were highly expressed in purple leaves, suggesting that *TGY009865.t1* may be involved in anthocyanin acylation and could contribute to anthocyanin transport in cooperation with MATE. The experiment did not identify any other DEGs involved in anthocyanin modification, which may indicate that the process of anthocyanin modification is not consistent within the population.

### *CsMYB75* is an important transcription factor for anthocyanin synthesis in purple samples

The R2R3-MYB transcription factors in tea plants are involved in flavonoid biosynthesis by activating the expression of structural genes, and they can also cooperate with WD40 and bHLH to form a ternary MBW complex to regulate flavonoid biosynthesis. Among them, CsMYB75 (CsAN1) is considered to be an important transcription factor in anthocyanin biosynthesis [27, 28]. Our experiment also confirmed the importance of this gene, which was the sole transcription factor highly expressed in the purple leaves of the population and exhibits variation on chromosome 2. Previous studies have identified *AN2* with a site mutation in the genetic process, leading to its inactivation in white flowers of petunia [29]. A recent study also revealed that the functional deficiency of *CsAN1* is the primary cause of the inability to accumulate anthocyanin, and the trait can be inherited by the offspring through hybridization in tea plants [30]. Furthermore, CsAN1 can interact with bHLH transcription factors and recruit CsTTG1 to form the MBW complex, which regulates anthocyanin accumulation [11]. This led us to next focus on the MBW complex, which may interact with *CsMYB75* in the purple leaves of this population.

Correlation analysis revealed that there are several genes significantly associated with *CsMYB75*, primarily belonging to the bHLH, MYB, IZIP, and other transcription factor superfamilies involved in anthocyanin synthesis. Among these, *TGY065369.t1* and *TGY030009.t1* showed a positive correlation with *CsMYB75*, for which a direct association with anthocyanin has not been documented. Only *RPP13*, as a homologous gene of *TGY030009.t1*, was found to be potentially associated with glutathione metabolism in maize under light conditions [31]. At this time, *TGY012529.t1*, a gene annotated as *CsMYB114-like*, negatively correlated with *CsMYB75* and located near *CsMYB75* on chromosome 2, aroused our curiosity.

Our research group has previously identified *CsMYB75* in ZJ through QTL mapping and transcriptome data and determined its role in anthocyanin production in *Arabidopsis* [13]. Due to the absence of a stable genetic transformation system and an effective transient expression system, previous studies on the function of genes in the tea plant only utilized heterologous expression systems such as *Arabidopsis* and tobacco. In recent years, transient systems for gene silencing and overexpression in tea plants have been reported with the advancement of research [32–34]. Thus, we silenced *CsMYB75* in the ZJ cultivar using antisense oligonucleotides, further confirming that *CsMYB75* can positively regulate the accumulation of anthocyanins in tea plants and significantly affect the structural genes related to anthocyanin synthesis and transport. However, the transcription factor gene *TGY012529.t1* showed a different expression compared with the earlier transcriptome, such that the expression of the gene decreased with the silencing of *CsMYB75*, indicating that it is not a competitive relationship as previously suspected. It was previously reported that this gene was localized in the nucleus, while it was mistakenly identified as *MYB75*, and its function was not verified [33]. In addition, it has been reported that the homologous gene of *MdMYB114* is unable to form the MBW complex in apples, but it can promote anthocyanin biosynthesis and transport by directly binding to the promoters of *MdANS*, *MdUFGT*, and *MdGST* [35]. Hence, it is still worth further studying whether the expression of this gene differs between purple and green leaves, or if there are other regulatory mechanisms.

Furthermore, how *CsMYB75* and its homologs are activated to control anthocyanin accumulation in plants is an important question to be answered. In red apple, Espley *et al.* found that an allelic rearrangement in the promoter of *MYB10*, a homolog of *CsMYB75*, has generated an autoregulatory locus that is sufficient to account for the expression increase of *MYB10* and subsequent ectopic accumulation of anthocyanins [36]. Self-activation of the MYB transcription factor in regulating anthocyanin accumulation is also reported in other plant species, such as cotton [37]. In this study, we also identified a 181-bp InDel in the *CsMYB75* promoter. The relative InDel marker co-segregated with leaf color (Fig. 6), indicating that the 181-bp insertion in the *CsMYB75* promoter is important for purple tea formation. The promoter prediction shows that the 181-bp insertion contains MYB transcription factor recognition and binding sites, suggesting the expression of *CsMYB75* might also be controlled by self-activation. Further investigation of the underlying mechanism will be beneficial to unravel the mystery of purple tea.

### GST-MRP and GST-MATE may be the common pathway of anthocyanin transport in purple samples

Anthocyanins are synthesized in the cytoplasm and then transported to the vacuoles for storage. Currently, the most complete mechanism involves the co-catalysis of cytoplasmic glutathione transferase (GST) and multi-drug resistance-related proteins (MRPs) in the vacuoles, whereas the primary transport mechanisms involve GST, MRP, MATE, and vesicles [38, 39]. The MRP subfamily is part of the ABCC superfamily, in which ABCC1 was identified as the transporter of anthocyanin-3-glucoside in grapes [40], and ABCC2 was found to transport anthocyanins and other flavonoids in *Arabidopsis* [41]. Rong *et al*. found that several miRNAs could target ABC transport genes and prevent apples’ anthocyanin from being transported from the cytoplasm to the vacuole [42]. In addition, Wei *et al.* identified CsGSTF1 linked to anthocyanin accumulation in ZJ [12]. It has been demonstrated that *CsGSTa*, *CsGSTb*, and *CsGSTc* are involved in the storage of anthocyanins, flavonols, and procyanidins in cells [43]. In our investigation, we found that *CsGSTa* is the DEG of *TGY013699.t1*, with high expression in purple leaves. Furthermore, numerous studies have shown that both MATE and ABCC may contribute to the transport of tea anthocyanins [12, 44, 45]. Taken together, our results show that in the purple bud one MATE gene and three ABCC genes were highly expressed, which may indicate that these genes are involved in transmembrane anthocyanin transport in the population of purple leaves. However, further research is necessary as the mechanism underlying intracellular flavonoid transport in tea plants is currently still unclear.

**Figure 7:**
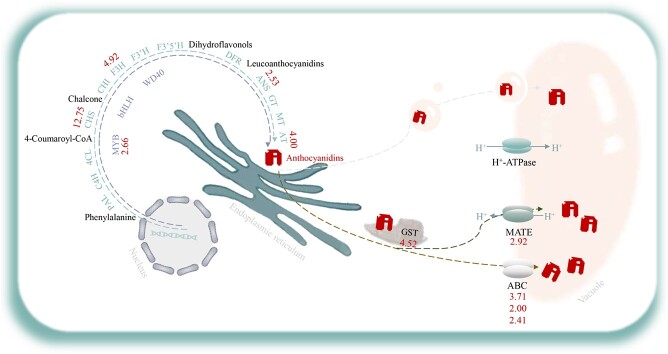
Schematic diagram of anthocyanin biosynthesis and transport in a purple population of tea plants. The numerical markers represent the expression of the gene in the purple sample, with the green sample as the control.

## Conclusions

In this study, RNA-seq, BSA-seq, and BSR-seq were utilized to identify the DEGs associated with anthocyanin content in purple samples, which is based on the genetic tea population with variations in leaf color. Ten genes, such as *CHS*, *F3H*, *ANS*, *MYB75*, *MATE*, and *ABCC*, were identified as potential participants in the purple formation of tea populations (Fig. 7), of which *CsMYB75* and *CsANS* were identified as key genes. Furthermore, it was shown that the *CsMYB75* regulates structural genes related to anthocyanin synthesis, thereby affecting anthocyanin content. Moreover, a 181-bp InDel in the *CsMYB75* promoter was identified to be co-segregating with leaf color. To our knowledge, this is the first time that the dual mixing pool method has been used to locate and study the anthocyanin synthesis pathway in purple populations, and, also for the first time, *CsMYB75* has been silenced in tea plants. Therefore, this study can provide a theoretical basis for further exploring the purple formation mechanism of tea plants and ultimately obtaining new tea cultivars with high anthocyanins.

## Materials and methods

### Plant materials

The tea plants were derived from the hybrid offspring *F*_1_ population of the purple tea cultivar ZJ and green tea cultivar JX, and were planted in Hangzhou, China. In the autumn of 2022, fresh leaves (one leaf and one bud) of 30 individuals with extreme leaf color (15 individuals including JX with totally green leaves, 15 individuals including ZJ with dark purple leaves) were selected and stored at −80°C. Five individuals of the same color were equally mixed into one tube, i.e. a total of six experimental materials (GP1, GP2, GP3, PP1, PP2, PP3), for RNA sequencing (Fig. 1A). In addition, we set up two sample pools, one for bulk segregant analysis sequencing (BSA-seq), denoted as GP and PP, and the other for bulked segregant RNA sequencing (BSR-seq), denoted as RGP and RPP. In the autumn of 2023, one bud and one leaf of ZJ were used in a functional identification experiment with *CsMYB75*. In the spring of 2024, one bud and one leaf of fathers for promoter cloning, and the offspring *F*_1_ population and some traditional cultivars (including the five purple leaf tea cultivars ZJ, ZZ1, QQM, YSH, and LZ7, and the five green leaf tea cultivars ZC108, WNZ, FDDB, ZM66, and ZM6) were used in an agarose gel electrophoresis experiment with *CsMYB75*.

### RNA-seq analysis

Using TRI Reagent (Qiagen, Valencia, CA; https://www.qiagen.com), total RNA was extracted from tea samples, and the TruSeq RNA Sample Prep Kit was used to purify the mRNA. Reverse transcriptase and six-base random primers were used to create the first- and second-strand cDNA. Following library synthesis, PCR and an Agilent 2100 Bioanalyzer were used to enrich fragments and detect the library, and the library was then combined, diluted, and denatured to create a single-strand library. Next, based on the Illumina platform, next-generation sequencing (NGS) was used to perform paired-end sequencing of libraries.

With the aid of Abraham Bioinformatics, the raw data were filtered. The resulting high-quality sequences were then mapped to the ‘Tieguanyin’ reference genome via the TopHat2 upgrade HISAT2 program. Gene expression level was computed based on the comparative results. Based on this information, expression difference analysis, cluster analysis, and enrichment analysis were used to further examine the samples. The criteria for DEGs were *P*-value <0.05 and |log_2_ (fold change)| > 1.

The RNA-seq raw data have been deposited in the NCBI SRA database (PRJNA935488).

### Methods used for BSA-seq and BSR-seq

Using Illumina’s TruSeq DNA PCR-free Preparation Kit (Illumina, https://www.illumina.com), BSA sequencing libraries were prepared. Initially, the ends of the retrieved DNA sequence were mended after ultrasonography randomly interrupted them. The kit’s End-Repair Mix2 repaired the missing bases at the 3′ end of the DNA sequence and eliminated the conspicuous bases at the 5′ end. We used 2% agarose gel electrophoresis, Beckman AMPure XP Beads, and PCR to enrich and purify the sequencing library fragments. The library was assessed and quantified using the Agilent High Sensitivity DNA Kit and the Quant-iT PicoGreen dsDNA Assay Kit. Following dilution, a single strand was denatured using NaOH, and 2 × 150 bp were paired-end sequenced using a NovaSeq sequencer. The BSR-seq library was constructed following the RNA-seq library.

Fastp (v0.20.0) and FastQC were used for quality control and data filtering of the raw data. Filtered reads were mapped using BWA (0.7.12-r1039) to the ‘Tieguanyin’ reference genome. SNPs and InDels were found and annotated using GenomeAnalysisTK v3.8 and ANNOVAR. After calculating the ED value, we screened for candidate regions linked to trait genes, and annotated the relevant candidate genes using GO, KEGG, etc.

The BSA-seq and BSR-seq raw data have been deposited in the NCBI SRA database (PRJNA1127093, PRJNA1127088).

### Quantitative real-time PCR analysis

qRT–PCR analysis was carried out in accordance with Chen *et al*. [46], utilizing SYBR Green dye (Sigma–Aldrich) and the ABI 7500 Real-Time PCR System with GAPDH as an internal control. Supplementary Data Table S10 contains a list of the primers used. The relative transcript levels were determined using the formula 2^−ΔΔCt^, and all results are shown as average ± standard deviation (*n* = 3).

### Anthocyanin content determination

With a little modification, a previously reported method was used to quantify the total anthocyanin content [12]. Five milliliters of acidic ethanol (0.1 mol/l ethanol hydrochloride) was used to extract 100 mg of the material for 30 min at 60°C. After that, the mixture was centrifuged for 5 min at 4000 rpm. After collecting the supernatant, the previous steps were carried out once more. A UV2550 spectrophotometer (Shimadzu, Japan) was used to test the extract’s absorbance at 520, 620, and 650 nm. The formula for calculating anthocyanin content was (*A* × *V* × *n* × 465.2)/(ε × m), where *A* is equal to (A530 − A620) − 0.1(A650 − A620).

### Suppression of CsMYB75 with AsODN inhibition in tea plants

The experimental methods refer to Zhao *et al.* [32] with a slight modification. AsODN candidate sequences of *CsMYB75* were designed using Soligo software (http://sfold.wadsworth.org), sense oligonucleotide (sODN) was used as a control, and nucleotide composition (40–60% GC) and oligonucleotide binding energy (≤ −8 kcal/mol) were used as selection criteria. To improve the interference effect, five sODN–AsODN pairs were synthesized and dissolved with ddH_2_O (Supplementary Data Table S11). 500 μl/20 μM AsODN and sODN solutions were injected into a 1.5-ml centrifuge tube, and one bud and one leaf of ZJ were cultured in an incubator for 1 and 4 days. Samples were taken and qRT–PCR was performed on *CsMYB75*, *ANS*, *CHS*, *F3H*, *GST*, and *TGY012529* (Supplementary Data Table S9).

### Amplification of *CsMYB75* promoter and development of marker

Primers were used to amplify the *CsMYB75* promoter sequence based on the genomic DNA of ‘Tieguanyin’ (Supplementary Data Table S12). The genome walking kit (Nanjing Vazyme Biotech Co., Ltd) served as the basis for the PCR settings, and 1% agarose gel electrophoresis was used to identify the PCR products; Hangzhou Youkang Biological Co., Ltd performed the sequencing.

## Supplementary Material

Web_Material_uhae191

## Data Availability

All data, tables, and figures in this manuscript are original, and are contained within the article and supplementary materials.
