# Supplementary material for: Association analysis of BSA-seq, BSR-seq, and RNA-seq reveals key genes involved in purple leaf formation in a tea population (Camellia sinensis)
Source: Hortic Res. 2024 Jul 10;11(9):uhae191. doi: 10.1093/hr/uhae191 (PMC11384119; doi:10.1093/hr/uhae191)
Supplement: Web_Material_uhae191 [file web_material_uhae191.zip › Supplementary Materials.doc]

Table S1 Disembarking data of RNA-Seq

| Sample | Reads No. | Bases (bp) | Q30 (bp) | N (%) | Q20 (%) | Q30 (%) |
| --- | --- | --- | --- | --- | --- | --- |
| PP1 | 47028510 | 7054276500 | 6627954149 | 0.000616 | 97.97 | 93.95 |
| PP2 | 46697168 | 7004575200 | 6554401919 | 0.000611 | 97.82 | 93.57 |
| PP3 | 44288606 | 6643290900 | 6238858513 | 0.000598 | 97.95 | 93.91 |
| GP1 | 43805636 | 6570845400 | 6240966321 | 0.00061 | 98.35 | 94.97 |
| GP2 | 42011640 | 6301746000 | 5916656140 | 0.000613 | 97.94 | 93.88 |
| GP3 | 43990992 | 6598648800 | 6246534525 | 0.000611 | 98.24 | 94.66 |

Table S2 RNA-Seq Map statistics

| Sample | Clean_Reads | Total_Mapped | Multiple_Mapped | Uniquely_Mapped |
| --- | --- | --- | --- | --- |
| GP1 | 41058814 | 37384302 (91.05%) | 1957357 (5.24%) | 35426945 (94.76%) |
| GP2 | 39532308 | 35984122 (91.02%) | 1856408 (5.16%) | 34127714 (94.84%) |
| GP3 | 41448690 | 38024044 (91.74%) | 1973619 (5.19%) | 36050425 (94.81%) |
| PP1 | 44205564 | 39892652 (90.24%) | 2129062 (5.34%) | 37763590 (94.66%) |
| PP2 | 43907174 | 39932937 (90.95%) | 2071418 (5.19%) | 37861519 (94.81%) |
| PP3 | 41611518 | 37660189 (90.50%) | 2000352 (5.31%) | 35659837 (94.69%) |

Table S3 BSA sequencing data statistics

| Sample | Reads Num | Total Bases (bp) | N (%) | GC (%) | Q20 (%) | Q30 (%) |
| --- | --- | --- | --- | --- | --- | --- |
| GP | 371587012 | 56109638812 | 0 | 40.46 | 97.34 | 93.06 |
| PP | 315964650 | 47710662150 | 0 | 40.45 | 97.27 | 92.98 |

Table S4 BSA high-quality data statistics

| Sample | HQ Reads | HQ Reads (%) | HQ Data (bp) | HQ Data (%) |
| --- | --- | --- | --- | --- |
| GP | 358640732 | 96.52% | 53113769089 | 94.66% |
| PP | 304889406 | 96.49% | 45089637252 | 94.51% |

Table S5 SNP/INDEL annotation result statistics of BSA-Seq

|  | SNP | | INDEL | |
| --- | --- | --- | --- | --- |
| Type | Number | Percentage | Number | Percentage |
| exonic total | 647079 | 1.27 | 13384 | 0.58 |
| synonymous SNV | 187672 | 0.37 | - | - |
| nonsynonymous SNV | 278982 | 0.55 | - | - |
| frameshift deletion | - | - | 3017 | 0.13 |
| frameshift insertion | - | - | 2399 | 0.1 |
| nonframeshift deletion | - | - | 2677 | 0.12 |
| nonframeshift insertion | - | - | 1934 | 0.08 |
| nonframeshift substitution | - | - | 0 | 0 |
| stopgain | 9615 | 0.02 | 142 | 0.01 |
| stoploss | 1219 | 0 | 50 | 0 |
| unknown | 169591 | 0.33 | 3165 | 0.14 |
| splicing | 0 | 0 | 0 | 0 |
| ncRNA total | 0 | 0 | 0 | 0 |
| ncRNA_exonic | 0 | 0 | 0 | 0 |
| ncRNA_exonic; splicing | 0 | 0 | 0 | 0 |
| ncRNA_splicing | 0 | 0 | 0 | 0 |
| ncRNA_intronic | 0 | 0 | 0 | 0 |
| ncRNA_UTR5 | - | - | 0 | 0 |
| intronic | 0 | 0 | 0 | 0 |
| intergenic | 47131268 | 92.29 | 1986128 | 86.62 |
| UTR5 | 0 | 0 | 29 | 0 |
| UTR3 | 0 | 0 | 0 | 0 |
| UTR5; UTR3 | 0 | 0 | 0 | 0 |
| upstream | 1337644 | 2.62 | 115037 | 5.02 |
| downstream | 1268993 | 2.48 | 109271 | 4.77 |
| upstream; downstream | 686034 | 1.34 | 69185 | 3.02 |
| Total | 51071018 | 100 | 2293034 | 100 |

Table S6 BSR sequencing data statistics

| Sample | Reads Num | Total Bases (bp) | N (%) | GC (%) | Q20 (%) | Q30 (%) |
| --- | --- | --- | --- | --- | --- | --- |
| RGP | 129808268 | 19471240200 | 0.00 | 46.39 | 98.18 | 94.52 |
| RPP | 138014284 | 20702142600 | 0.00 | 45.49 | 97.91 | 93.81 |

Table S7 BSA high-quality data statistics

| Sample | HQ Reads | HQ Reads (%) | HQ Data (bp) | HQ Data (%) |
| --- | --- | --- | --- | --- |
| RGP | 126664798 | 97.58% | 18797693933 | 96.54% |
| RPP | 134283326 | 97.30% | 19908283475 | 96.17% |

Table S8 SNP/INDEL annotation result statistics of BSR-Seq

|  | SNP | | INDEL | |
| --- | --- | --- | --- | --- |
| Type | Number | Percentage | Number | Percentage |
| exonic total | 242775 | 23.06 | 6720 | 6.9 |
| synonymous SNV | 85943 | 8.16 | - | - |
| nonsynonymous SNV | 90306 | 8.58 | - | - |
| frameshift deletion | - | - | 765 | 0.79 |
| frameshift insertion | - | - | 911 | 0.94 |
| nonframeshift deletion | - | - | 1893 | 1.94 |
| nonframeshift insertion | - | - | 1616 | 1.66 |
| nonframeshift substitution | - | - | 0 | 0 |
| stopgain | 783 | 0.07 | 38 | 0.04 |
| stoploss | 169 | 0.02 | 28 | 0.03 |
| unknown | 65574 | 6.23 | 1469 | 1.51 |
| splicing | 0 | 0 | 0 | 0 |
| ncRNA total | 0 | 0 | 0 | 0 |
| ncRNA_exonic | 0 | 0 | 0 | 0 |
| ncRNA_exonic; splicing | 0 | 0 | 0 | 0 |
| ncRNA_splicing | 0 | 0 | 0 | 0 |
| ncRNA_intronic | 0 | 0 | 0 | 0 |
| ncRNA_UTR5 | - | - | 0 | 0 |
| intronic | 0 | 0 | 0 | 0 |
| intergenic | 414661 | 39.38 | 40971 | 42.09 |
| UTR5 | 0 | 0 | 14 | 0.01 |
| UTR3 | 0 | 0 | 0 | 0 |
| UTR5; UTR3 | 0 | 0 | 0 | 0 |
| upstream | 123359 | 11.72 | 19310 | 19.84 |
| downstream | 137166 | 13.03 | 16856 | 17.32 |
| upstream; downstream | 134901 | 12.81 | 13467 | 13.84 |
| Total | 1052862 | 100 | 97338 | 100 |

Table S9 Cis-acting elements analysis of 181bp InDel in 'ZJ'

| Function | Motifs | Position | Sequence |
| --- | --- | --- | --- |
| Hormonal responsive | CAATBOX1 | 4 | CAAT |
| Hormonal responsive | ARR1AT | 7 | NGATT |
| Protein storage responsive | SEF4MOTIFGM7S | 20 | RTTTTTR |
| Oxidative stress | GATABOX | 26 | GATA |
| Abiotic and biotic stress | GTGANTG10 | 28 | GTGA |
| Hormonal responsive | CPBCSPOR | 34 | TATTAG |
| Hormonal responsive | CAREOSREP1 | 82 | CAACTC |
| Abiotic and biotic stress | GTGANTG10 | 101 | GTGA |
| MYB binding site | MYB2CONSENSUSAT | 110 | YAACKG |
| MYB binding site | MYBCORE | 110 | CNGTTR |
| MYB binding site | MYBCOREATCYCB1 | 111 | AACGG |
| Light responsive | SORLIP1AT | 127 | GCCAC |
| Light responsive | BOXCPSAS1 | 139 | CTCCCAC |
| Light responsive | GT1CONSENSUS | 168 | GRWAAW |
| Salt/pathogen responsive | GT1GMSCAM4 | 168 | GAAAAA |
| Pollen-specific activation-related | POLLEN1LELAT52 | 170 | AGAAA |

Table S10. Primers used for quantitative real time PCR analysis

| Gene ID | Forward Primer (5' - 3') | Reverse Primer (5' - 3') |
| --- | --- | --- |
| TGY009865.t1 | CGATGTGTTTCTCGGCACTTTT | ACTTTCTGGCTCCTCGTTTTCA |
| MYB75 (TGY012519.t1) | TGCTGGGTCTTGCTCTCTTTCT | CTTTCACCTTTTCTCTTTGGGT |
| TGY019907.t1 | TGCTGGGTCTTGCTCTCTTTCT | AATTGGTGATTCTAGGTTTCCG |
| TGY029095.t1 | AATCCACTCTATCCTCCCTAAC | GATCCCTATACTCACCCGTAAC |
| TGY037912.t1 | GAAGTTGGATGGTTCGATGAGG | AGGACAAGAAGAGGGAAGGTGG |
| TGY072372.t1 | AGAGCTCAAGGTGAAGGAGATC | CTGGTACAAAGTTTGTGGCATA |
| TGY084885.t1 | GCTGTTGATTTCCTCCTCCTTG | GGGCGGCATACTCTAACTTCTT |
| GAPDH | TTGGCATCGTTGAGGGTCT | CAGTGGGAACACGGAAAGC |
| ANS (TGY116873.t1) | TGCGTCCCCAACTCCATTATCA | GGCTCCTCCTCCGTCACTGTCT |
| F3H (TGY116395.t1) | GCTGTCCATAGCTAGTTTTCAGAGT | GTACCCTTTTGGGTGCTCTTC |
| CHS (TGY014300.t1) | GCGAGCATAAGACCGAACTC | ACCAAGTGGGTGATTTTGGA |
| GST (TGY013699.t1) | AAGTGTATGGTCCAGTTAGGGC | AGTAAGAAATCGGGTTTTTTAT |
| TGY012529.t1 | AAAGGTGCATGGACTGAGGA | TGAAAGGAACTTGGTGCCACT |

Table S11. Primers for CsMYB75 suppression of tea

| sODN (5' - 3') | AsODN (5' - 3') |
| --- | --- |
| GAAAATAAGACCAGGTACAC | GTGTACCTGGTCTTATTTTC |
| GGAAAATAAGACCAGGTACA | TGTACCTGGTCTTATTTTCC |
| GGGAAAATAAGACCAGGTAC | GTACCTGGTCTTATTTTCCC |
| ACCACCATCGCGGGATGATG | CATCATCCCGCGATGGTGGT |
| TACCACCATCGCGGGATGAT | ATCATCCCGCGATGGTGGTA |

Table S12. Primers for CsMYB75 promoter cloning

|  |  | Forward Primer (5' - 3') | Reverse Primer (5' - 3') |
| --- | --- | --- | --- |
| P1 |  | ATGTGCCTCTTCCAATCCTGTC | ATGGAACACAGCAAACACTGTCC |
| P2 |  | CACACCGCATAAATTTAATCACA | CACCTTTTCTCACTCCTAATGGA |

Note: Primer P1 was used for promoter cloning of CsMYB75 in 'ZJ' and 'JX', and primer P2 was used for PCR amplification of CsMYB75 in gel electrophoresis of F1 population.

Figure S1. Classification statistics of leaf color in F1 population
